# A Novel Balloon Catheter-based Dilation Intervention for Patients with Cricopharyngeus Achalasia After Stroke: A Randomized Study

**DOI:** 10.1007/s00455-021-10400-6

**Published:** 2022-01-27

**Authors:** Tingting Hu, Yeyu Cai, Zhaohui Shen, Ailian Chen, Yacen Wu, Tao Song, Jia Liu, Chujuan Liu, Fanghua Gong

**Affiliations:** 1 Hunan Provincial People’s Hospital, (The First Affiliated Hospital of Hunan Normal University), Changsha, 410002 People’s Republic of China; 2 The Second Xiangya Hospital of Central South University, Changsha, 410011 People’s Republic of China; 3 Changsha No.1 Social Welfare Institute, Changsha, 410021 People’s Republic of China

**Keywords:** Cricopharyngeus achalasia, Stroke, Dilation, Balloon catheter, Ordinary urinary catheter

## Abstract

This study aimed to investigate the efficacy and safety of a novel balloon catheter in dilation intervention for patients with cricopharyngeus achalasia after stroke. Thirty-four patients with cricopharyngeus achalasia after stroke received routine swallowing rehabilitation training and were randomly assigned to an experimental group (Exp, *n* = 17) that received dilation therapy using the novel balloon catheter once daily for 5 days per week or a control group (Con, *n* = 17) that received dilation therapy with a 14-Fr ordinary urinary catheter once daily for 5 days per week. The intervention duration, Eating Assessment Tool (EAT)-10 scores, and Functional Oral Intake Scale (FOIS) scores were recorded at baseline and each day during intervention. The time for a patient’s FOIS score to be ≥ 3 as well as the recovery time for oral intake of water, liquid food, mushy food, and solid food were recorded or estimated. Complications were also recorded during intervention. The intervention duration was shorter in the Exp group than in the Con group (*p* = 0.005). The Exp group patients improved faster than the Con group patients, with a shorter recovery time for oral intake of liquid food (*p* = 0.002), mushy food (*p* = 0.001), and solid food (*p* = 0.001). At the time of intervention termination, EAT-10 scores were lower in the Exp group than in the Con group (*p* = 0.005). The Exp group had a similar incidence of complications as the Con group but with better tolerability (*p* = 0.028). Compared with the urinary catheter, the novel balloon catheter for dilation in patients with cricopharyngeus achalasia after stroke may lead to a better and more rapid recovery.

## Introduction

Cricopharyngeus achalasia is a common cause of dysphagia caused by failure of relaxation of the cricopharyngeus muscle during the pharyngeal phase of swallowing [1–3]. Its clinical symptoms and complications include dysphagia, coughing, choking, nasopharyngeal regurgitation, aspiration pneumonia, weight loss, and malnutrition [4, 5]. Cricopharyngeus dysfunction (CPD) can be a consequence of a spectrum of neurological diseases, including stroke, multiple sclerosis, amyotrophic lateral sclerosis, primary muscle disorders, skull base neoplasia, and Parkinson’s disease [3]. Patients with stroke have a relatively high risk of cricopharyngeus achalasia, especially in cases of brain stem stroke, which has an incidence rate of up to 70% [6].

A range of management stratagies exist to improve the cricopharyngeus muscle function and swallowing, including compensatory strategies, rehabilitation exercises, physical dilation, pharmacological interventions, and surgery. Dilation is the most commonly used intervention and can include bougies, a wire-guided polyvinyl dilator, air-filled pneumatic dilation, and water-filled balloon dilation with or without endoscopy guidance [7, 8]. Among these, balloon catheter dilation is the most effective method, which has been investigated in the previous study [9]. A previous study has demonstrated good efficacy with urethral catheter dilation in CPD patients after stroke, with both active and passive balloon dilation presenting benefits (narrowing or obstruction disappeared rate of 76.2% vs. 76.5%, respectively) [10]. However, to the best of our knowledge, balloon catheter dilation protocols have not been standardized yet. Although urethral catheters (No.14-No.16) are commonly used in China, these balloons are oval and small with several disadvantages such as heterogeneous pressure distribution, short ice stimulation time, and small expansion range.

In this study, we developed a novel balloon dilation catheter by improving the tube material, internal circulation structure, balloon shape, and ordinary urinary catheter length. This study aimed to investigate the effectiveness of dilation intervention using our novel balloon dilation catheter in patients with cricopharyngeus achalasia after stroke.

## Methods

This study protocol was approved by the Ethics Committee of Hunan Provincial People's Hospital (No. 2021019). Informed consent was obtained from each participant for study inclusion.

### Design

This is a randomized controlled trial with concealed allocation, intention-to-treat analysis, and blinded assessment. The study was performed at the Department of Rehabilitation of Hunan Provincial People's Hospital, Changsha, Hunan, China. Patients at this hospital with cricopharyngeus achalasia after stroke who required dilation therapy were invited to participate in the study. Eligible and willing participants received routine swallowing training and nursing care and were randomly divided into an experimental group (treated with the novel balloon dilation catheter; Exp group) and a control group (treated with a No.14 ordinary urinary catheter; Con group) in a 1:1 ratio. Baseline characteristics such as age, sex, dysphagia cause, comorbidities, and disease course were recorded for each participant.

Concealed allocation of the randomization was performed using a computer-generated random sequence and opaque envelopes by a researcher who was not involved in this study. Only the physiotherapist who administered the intervention to each patient was not blinded to the research information. Another rehabilitation physician who was blinded to the information measured the outcomes.

Dilation was performed by a rehabilitation physician after baseline measurements were recorded and was continued once daily for 5 days per week. Eating Assessment Tool (EAT)-10 score, Functional Oral Intake Scale (FOIS) score, ability of oral intake, and complications were assessed each day during the intervention by another blinded rehabilitation physician. This assessment physician decided when the dilation therapy should be terminated. If the swallowing function recovered prior to completing the intervention protocol period, outcomes were measured after the last dilation intervention to homogenize the analyses.

### Participants

Patients with cricopharyngeus achalasia after stroke who required dilation therapy at the hospital were recruited, screened for eligibility, and confirmed to willingly participate in this study from March 2019 to December 2020. The inclusion criteria were as follows: 1) diagnosis of cricopharyngeus achalasia confirmed by videofluoroscopic swallowing study (VFSS) or clinical evaluation; 2) stroke confirmed by radiologists based on head magnetic resonance imaging; 3) age 20–80 years; and 4) meets the indication of dilation therapy and unwilling to undergo conventional swallowing therapies, including surgery. The excludion criteria were as follows: 1) organic obstruction of nasal or pharyngeal passage; 2) sever heart, lung, or kidney disease; 3) mental illness; and 4) severe cognitive disorder or aphasia. According to VFSS, cricopharyngeus achalasia is defined as the presence of complete or partial failure of cricopharyngeal relaxation, as observed by a radiologist [11]. Clinical evaluation was performed based on the clinical swallow evaluation standardized protocol, also known as clinical non-instrumental evaluation, which consists of anamnesis evaluation, morphodynamic evaluation, and an oral feeding test [12].

### Intervention

All the participants underwent routine swallowing rehabilitation training, including basic swallowing training (oral and facial function training and Shaker training, once daily for 30 min), oral intake training, and VitalStim electrical stimulation (once daily for 30 min). Nutritional and mental care was provided to all participants.

#### Experimental Group

Patients in this group received dilation therapy using the novel balloon dilation catheter accordingly to the following steps.

##### Novel Balloon Dilation Catheter

**Fig. 1:**
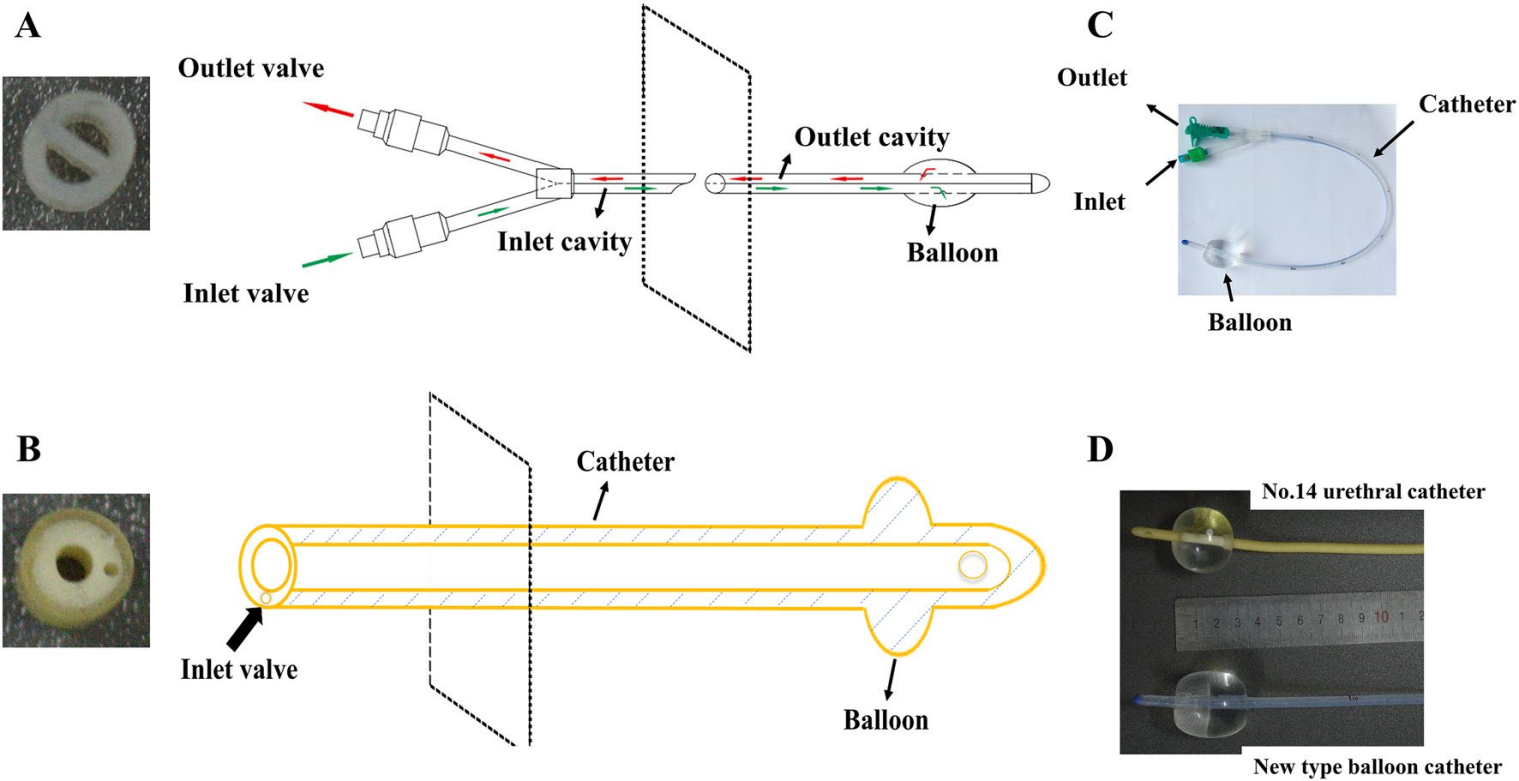
Image and rendering of the novel balloon catheter (**A**, **C**) and No.14 standard urinary catheter (**B**). The size and shape of the two types of balloon (**D**)

This novel balloon dilation catheter comprises a Y-shaped connector with inlet and outlet valves, a catheter body, and a balloon (Fig. 1C). The catheter body is divided into two cavities (inlet cavity and outlet cavity), which are connected to the inlet and outlet valves, respectively. Both the cavities are connected to the balloon. Water is injected from the inlet valve, which then flows through the inlet cavity to the balloon, passes through the balloon to the outlet cavity, and then flows out from the outlet valve (Fig. 1A). There are several advantages to this novel balloon dilation catheter.

1. In contrast to the No.14 ordinary urinary balloon catheter (Fig. 1B), which only has a single cavity, this novel balloon catheter with double cavities separates the inlet and outlet water flow to ensure that the water can flow continuously and evenly in and out of the catheter. Double cavities increase the ice stimulation time that benefits the sensitivity of the swallowing reflex areas.
2. Compared with the urinary catheter’s perfectly spherical-shaped balloon with a maximum length of 2 cm, the novel balloon dilation catheter’s ellipsoid-shaped balloon with a length of 4 cm (Fig. 1D) is conducive to more adequate expansion of the cricopharyngeal muscle.
3. When fully inflated, the novel balloon can be expanded larger compared with a urethral catheter balloon with radial sizes of 3 cm vs. 2 cm, respectively. (Fig. 1D).
4. The catheter is marked with a scale, so that the operator can clearly read the depth of catheter insertion.

##### Dilation Steps

Step 1: Patients were asked to fast for 2 h before dilation. The catheter was gently inserted into the patient's nasal cavity (or oral cavity when a patient's deglutition reflex was not present) after confirming its integrity. Simultaneously, patients were instructed to swallow. When the catheter was completely inserted into the esophagus (about 30 cm in length from the nasal cavity), patients were asked to make sounds. If the patient sounded normal without coughing, the operator proceeded to the next step.

Step 2: After the catheter was fully inserted into the esophagus, the inlet and outlet valves of the Y-connector were opened, and ice water was injected continuously into the inlet cavity using a syringe. The ice water remained flowing in and out for 3–5 min.

Step 3: The outlet valve was closed, and 5–7 mL of ice water was injected to fill the balloon. Then, the inlet valve was closed.

Step 4: The operator could then pull out the catheter gently. When the balloon passed through the cricopharyngeal muscle (the position where the catheter was hard to pull out), the balloon size was adjusted by altering the water volume in the balloon to ensure that the operator could only feel slight resistance. Simultaneously, the operator pulled out the balloon slowly and repeatedly while instructing the patient to swallow until the balloon left the esophagus.

Step 5: After the balloon was removed from the esophagus, the outlet valve was opened to drain the water. Then, the catheter could be pulled out completely.

Step 6: The above steps were repeated 5–6 times, with the whole treatment lasting for approximately 30 min.

Note: The balloon volume was increased by 1–2 mL daily, with total amount of < 20 mL.

#### Control Group

Patients in this group received dilation therapy using a No.14 ordinary urinary catheter with the following steps.

Step 1: The same as that for the experimental group.

Step 2: After the catheter was completely inserted into the esophagus, 1–2 mL of ice water was injected into the balloon, and the inlet valve was closed.

Step 3: The operator pulled out the catheter gently until the balloon left the esophagus, while instructing the patient to swallow. The specific process was the same as that for the experimental group.

Step 4: After the balloon left the esophagus, the ice water was withdrawn from the balloon with a syringe, and the catheter was pulled out completely. Subsequently, the remaining water was drained, and the above steps were repeated 5–6 times. The entire process lasted for approximately 30 min.

Note: The balloon volume was increased by 1–2 mL daily, with total amount of < 15 mL.

### Outcome Measures

All the participants were assessed at baseline and every day during the intervention period by a rehabilitation physician blinded to this study. The primary outcome was the dilation duration, which was quantified in days, until recovery of the swallowing function, which was assessed by the physician based on the criteria of a FOIS score of ≥ 6. Swallowing function was assessed using both the FOIS [13] and EAT-10 scoring systems [14]. The EAT-10 scoring system comprises 10-item questions, including various dysphagia symptoms, clinical characteristics, psychological feelings, and social influence. Each question is divided into 5 levels: none (0), mild (1), moderate (2), severe (3), and extreme (4). A final total EAT-10 score of ≥ 3 is considered abnormal. The FOIS score categorizes oral intake into 7 grades from I to VII, corresponding to 1 to 7 points. An FOIS score of ≥ 3 implies that the clinical outcome is significantly improved. Oral intake training was conducted every day during the intervention period by the physician using one-bite size of water, liquid food, mushy food, or solid food. According to the water swallow test, recovery of oral intake of water was defined as swallowing at once without coughing. During the intervention period, complications, such as laryngeal edema and mucosal hemorrhage, were evaluated in all the patients by the rehabilitation physician and the senior nurse who were blinded to the study. Pain status was assessed using a five-point oral grading scale [15] (0: no pain, 1: mild pain, 2: moderate pain, 3: severe pain, 4: extreme pain, and 5: intolerable pain) by the nurse.

### Data Analysis

Sample size was calculated based on the primary outcome of intervention duration. A mean duration of 8 days for the experimenal group and 16 days for the control group, with a standard deviation of 10 and 2, respectively, was anticipated, and a significance level of 5% and study power of 86% were used. Finally, a sample of 30 participants (15 per group) was required. In total, a sample size of 34 participants was used to allow for possible participant or data loss.

All data analyses were performed by a researcher who was not involved in this study assessments and interventions. Baseline characteristics of the participants were summarized with descriptive statistics. Statistical analysis was performed following the intent-to-treat principle. The Kruskal–Wallis one-way analysis of variance test was used for continuous variables, including the EAT-10 score and recovery time for oral intake of water, liquid food, and mushy food. Due to the non-normal distribution of data, the between-group differences in dilation duration and recovery time for oral intake of solid food were investigated using the Mann–Whitney U-test. The chi-square test was used for categorical variables such as complications. A significance level of 5% (*p* < 0.05) was used for all statistical analyses. All statistical analyses were performed using SPSS software (IBM).

## Results

### Flowchart of Participants Through the Study

**Fig. 2:**
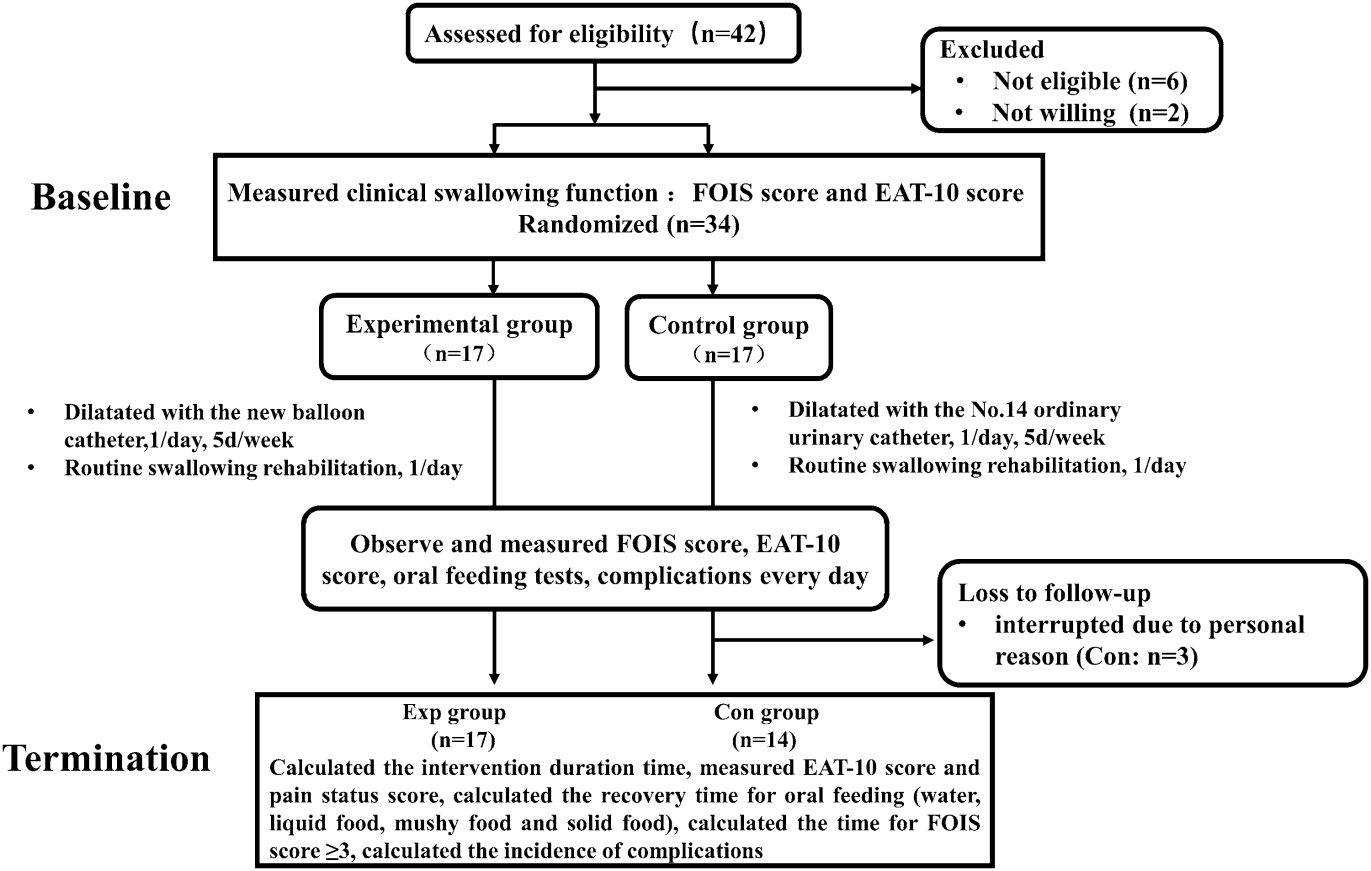
Design and flowchart of the study participants

A total of 40 consecutive patients with cricopharyngeus achalasia after stroke were evaluated for eligibility from March 2019 to December 2020. Among these, 9 patients were excluded for reasons stated in Fig. 2. The remaining 34 participants were eligible and willing to participate in the study and were randomized into the experiemental (Exp) (*n* = 17) and control (Con) (*n* = 17) groups. All the participants received the correct designated intervention. The treatment was interrupted in 3 participants in the Con group who were discharged during intervention due to personal reasons unrelated to the study. None of the physicians or nurses was unblinded to information during the study. In total, measurements were recorded and analyzed for 17 participants in the Exp group and 14 participants in the Con group.

### Characteristics of the Participants

According to the baseline characteristics (Table 1), there were no significant differences in sex, age, disease course, and combinations (hypertension, diabetes and coronary heart diseases) between the two groups.

**Table 1:** Participant demographic and baseline characteristics

Characteristics	Exp (*n* = 17)	Con (*n* = 17)	*p* Value
Age (y), median (ranging from)	60 (49 to 77)	55.5 (43 to 77)	0.420
Sex, *n* male (%)	15 (88)	11 (69)	0.112
Cause of dysphagia, *n* (%)			
Infarction	15 (88)	14 (88)	1.000
Hemorrhage	2 (12)	2 (12)	1.000
Comorbidities, *n* (%)			
Hypertension	13 (76)	13 (76)	1.000
Diabetes	5 (29)	4 (25)	1.000
Coronary heart diseases	4 (24)	2 (12)	0.659
Course of dysphagia (d), median (ranging from)	21 (7 to 90)	26 (9 to 60)	0.504
FOIS, median (ranging from) (0 to 7)	1 (1 to 4)	1.5 (1 to 3)	0.978
EAT-10, median (ranging from) (0 to 40)	29 (15 to 36)	28 (11 to 36)	0.261

*Exp* experimental group (using the novel balloon catheter dilation), *Con* control group (using the ordinary urinary catheter dilation), *FOIS* functional oral intake scale, *EAT-10* eating assessment tool-10

### Effects of the Intervention

#### Dilation Duration

The total intervention duration ranged from 5 to 25 days, with a duration of 5–15 days in the Exp group and 6–25 days in the Con group. The dilation duration was significantly shorter in the Exp group than in the Con group (9 vs. 14 days, *p* = 0.005) (Table 2).

**Table 2:** Effects and complication measurements between groups

Outcomes	Group		*p* value
	Exp (*n* = 17)	Con (*n* = 14)	
Duration of dilation (d), median (ranging from)	9 (5 to 15)	14 (6 to 25)	0.005
EAT-10 score at termination, median (ranging from)	3 (2 to 6)	5 (2 to 8)	0.005
Recovery time of FOIS of 3 or more (d), median (ranging from)	4 (1 to 6)	5 (3 to 15)	0.030
Recovery time for oral intake of (d), median (ranging from):			
Water	5 (1 to 10)	5 (3 to 20)	0.426
Liquid food	4 (1 to 8)	8 (4 to 20)	0.002
Mushy food	5 (3 to 10)	10 (5 to 25)	0.001
Solid food	7 (4 to 15)	13 (6 to 25)	0.001
Complications (*n*.%)			
Mucosal bleeding	1 (5.9%)	4 (28.6%)	0.148
Laryngeal edema	1 (5.9%)	3 (21.4%)	0.304
Pain (0–5), median (ranging from)	0 (0 to 1)	1 (0 to 2)	0.028

*Exp* experimental group (using the novel balloon catheter dilation), *Con* control group (using the ordinary urinary catheter dilation). *FOIS* functional oral intake scale, *EAT-10* eating assessment tool-10

#### EAT-10 Score

Both the groups showed significantly improvements in EAT-10 scores at the end of the intervention. At the time of intervention termination, the EAT-10 scores were significantly lower in the Exp group than in the Con group (3 vs. 5, *p* = 0.005) (Table 2).

#### Recovery Time for Oral Intake

**Fig. 3:**
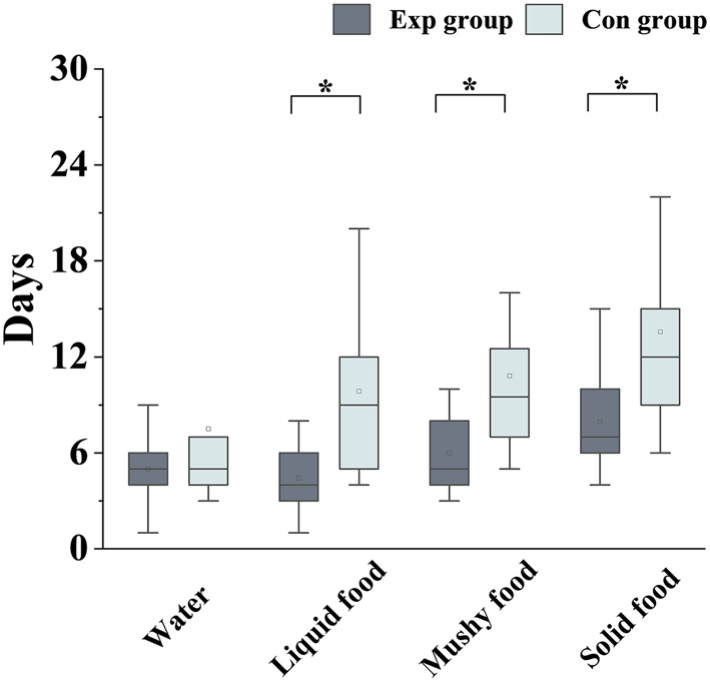
Bar chart of comparison between Exp group and Con group in the recovery time of oral intake of water, liquid food, mushy food and solid food. *p < 0.05

All the participants achieved complete recovery for oral intake of water and three different food types (liquid food, mushy food, and solid food). The recovery time for oral intake of liquid food (*p* = 0.002), mushy food (*p* = 0.001), and solid food (*p* = 0.001) was significantly shorter in the Exp group than in the Con group (Table 2, Fig. 3). However, the recovery time for oral intake of water was similar between the Exp and Con groups (5 vs. 5 days, *p* = 0.426).

#### Complications

During intervention, the incidence of mucosal bleeding (5.9% vs. 28.6%, *p* = 0.148) and laryngeal edema (5.9% vs. 21.4%, *p* = 0.304) was similar between the Exp and Con groups. Regarding tolerability, 4 patients (23.5%) in the Exp group experienced pain with a median pain score of 0, whereas 8 patients (57.1%) in the Con group experienced pain with a median score of 1. Patients in the Exp group had significantly better tolerability than patients in the Con group (*p* = 0.028), as shown in Table 2.

## Discussion

This study determined that the use of the novel balloon catheter for dilation was more beneficial than the use of an ordinary urinary catheter in terms of several outcomes, including shorter dilation duration, shorter recovery time for swallowing function, and shorter recovery time for oral intake of food. Compared with the ordinary urinary catheter, this novel balloon catheter did not result in a higher complication incidence but had better tolerability.

To the best of our knowledge, this is the first study wherein a novel balloon dilation catheter is used in patients with cricopharyngeus achalasia after stroke. We believe that there are two reasons why the novel catheter can help patients recover more rapidly compared with an ordinary urinary catheter. First and most importantly is ice stimulation, which is an effective method to improve the motor and sensory activities of the tongue and soft palate through repeated mechanical, temperature, and pressure stimulation [16]. It increases the sensitivity of the swallowing reflex areas by stimulating the sensory input nerve fibers and eventually induces the swallowing reflex to improve the swallowing function [17, 18]. Kawakami,M et al*.* first reported the excitability of the human cortical swallowing motor pathway after oral ice stimulation and found that oral ice stimulation increases the excitability of the corticobulbar projection to the mylohyoid muscles, but ice stimulation to the superficial neck skin does not have any effects [19]. The No.14 ordinary urinary catheters, which are commonly used in clinical practice, have a balloon with only one water inlet valve. Filling the balloon with ice water and inserting it into the esophagus can achieve a relatively short time of ice stimulation, as the temperature rises quickly. To extend the ice stimulation time, we developed this new catheter with two independent cavities, both connected to the balloon, which could achieve continuous circulation of ice water in and out of the catheter. Thus, the cricopharyngeus muscle could continuously receive constant low temperature ice stimulation. Therefore, we believe that the combination of physical dilation and a relatively long-term ice stimulation has more beneficial effects compared with the combination of physical dilation and a relatively short-term ice stimulation.

In addition, the shape and size of the balloon might be the second reason for the superiority of our novel catheter. The cricopharyngeus muscle is the main muscle of the upper esophageal sphincter, which is a high-pressure zone lying between the pharynx and the cervical esophagus. Lang et al*.* [20] and Gerhardt et al*.* [21] demonstrated that the upper esophageal sphincter comprises the proximal esophageal circular muscle, cricopharyngeus muscle, and thyropharyngeal muscle, and the pressure gradually increases in this order. Sivarao et al*.* compared the manometric data from three different sources of the upper esophageal sphincter and concluded that the peak high-pressure zone lies above the anatomical extent of the cricopharyngeus muscle [22]. Although the cricopharyngeus muscle is only 1–2 cm wide, the high-pressure zone may be 2.5–4.5 cm in length [23]. Thus, patients with cricopharyngeus achalasia require dilation not only for the cricopharyngeus muscle but also for the entire high-pressure upper esophageal sphincter zone. The ordinary urinary catheter, which is only 2 cm in length, will cause insufficient expansion in this regard. Therefore, by increasing the length of the balloon to 4 cm, the upper esophageal sphincter high-pressure zone can be fully dilatated.

Despite the recovery time for oral intake of different foods, including liquid food, mushy food, and solid food, being shorter in the Exp group, the recovery time for oral intake of water was not markedly different between the two groups. Mostly likely, this is because the definition of recovery for oral intake of water involved drinking without choking. There are many other reasons for choking during drinking, besides cricopharyngeus muscle dysfunction, especially in patients with stroke. Therefore, even when the cricopharyngeus muscle function recovers, patients may still choke during drinking water for a prolonged period.

Despite the total time required for each intervention being similar between the two groups, i.e. approximately 30 min per intervention, the use of the novel balloon catheter was more easy comapred with the use of the ordinary urinary catheter; however, a longer time was taken for ice stimulation in the Exp group, eventually resulting in similar times for both the catheters. We believe there is a benefit in the novel catheter being made from medical silicone rather than latex, similar to the urinary catheters. A stiffer catheter makes it easier to pass through the high-pressure zone of the upper esophageal sphincter. Simultaneously, due to the single inlet valve of the ordinary urinary balloon catheter, the syringe needs to be used multiple times during operation, which increases the time and difficulty of the procedure.

Regarding adverse events in our study, the incidence of mucosal bleeding and laryngeal edema and the pain scores were similar between both the groups. In a study by Wu et al*.* [24], minor self-limiting mucosal bleeding was commonly observed during dilation but neither endoscopic hemostasis or blood transfusion was required. These results show that this type of intervention is a relatively safe and tolerable method for patients with cricopharyngeus achalasia. The novel balloon catheter is also as safe as the ordinary urinary catheter.

In our study, the physician who was blinded to the study decided whether the dilation therapy should be terminated or not based on the FOIS scores. The FOIS scoring system is a 7-point ordinal scale that reflects the functional oral intake [25]. Crary,M. A et al*.* have demonstrated that the FOIS scoring system is an independent measure of functional oral intake in a prospective study [26]. In our study, intervention was terminated when the FOIS score of participants was ≥ 6, which indicates normal oral intake without special requirements with or without special diet restrictions. This is also used in ordinary clinical practice as one of the discharge criteria for patients with dysphagia. According to Huai et al*.,* the clinical outcomes of patients with dysphagia were considered to be significantly improved when their FOIS score increased to ≥ 3 [5]. In our study, the recovery time for an FOIS score of ≥ 3 in the Exp group was shorter than that in the Con group, indicating that the clinical outcomes improved quicker when patients received the novel balloon catheter dilation. The EAT-10 scoring system is a 10-item self-administered questionnaire about the severity of dysphagia symptoms and its clinical and social impact [27]. A previous study has demonstrated that a final EAT-10 score of ≥ 3 is considered abnormal [14]. In our study, the mean EAT-10 score at time of treatment termination in the Exp group (3) was significantly lower than that in the Con group (5). From these results, we can conclude that not all participants had an EAT-10 score of < 2 when the intervention was terminated. We believed the reason why the FOIS score recovered to normal but the EAT-10 score did not may be to do with psychological feelings and social influences. Although some patients achieved complete recovery for oral intake of water and foods, they still maintained some psychological barriers.

There are still some limitations of our study. First, this study used the EAT-10 and FOIS scores to clinically evaluate the swallowing function, whereas in future studies we plan to use gold standards methods such as VFSS or videoendoscopy swallowing study. Second, this study focused on patients with dysphagia after stroke, but the dysphagia degree and recovery ability vary according to the location and distribution of the stroke. In future studies, we will conduct subgroup analysis of patients with brain stroke in different locations. Third, the adverse events, including laryngeal edema or mucosal bleeding, in our study were assessed through visual evaluation by a physician. In future studies, we aim to use more accurate methods of evaluation, such as laryngoscopy. Finally, due to the time limitation, only short-term efficacy was discussed in this study, the long-term efficacy through follow-up examination will be discussed in a future study.

In conclusion, dilation intervention using the novel balloon catheter improves the swallowing function of patients with cricopharyngeus achalasia after stroke in a more effective and faster manner compared with the ordinary urinary catheter. This novel balloon catheter can be recommended for clinical practice.
